# Optimization of Alkaline Extraction and Bioactivities of Polysaccharides from Rhizome of *Polygonatum odoratum*

**DOI:** 10.1155/2014/504896

**Published:** 2014-07-01

**Authors:** Yong Chen, Luoyi Yin, Xuejiao Zhang, Yan Wang, Qiuzhi Chen, Chenzhong Jin, Yihong Hu, Jihua Wang

**Affiliations:** ^1^Department of Life Sciences, Hunan Institute of Humanities, Science and Technology, Loudi 417000, China; ^2^Crop Research Institute, Guangdong Academy of Agriculture Sciences, Guangzhou 510640, China

## Abstract

The present study is to explore the optimal extraction parameters, antioxidant activity, and antimicrobial activity of alkaline soluble polysaccharides from rhizome of *Polygonatum odoratum*. The optimal extraction parameters were determined as the following: NaOH concentration (A) 0.3 M, temperature (B) 80°C, ratio of NaOH to solid (C) 10-fold, and extraction time (D) 4 h, in which ratio of NaOH to solid was a key factor. The order of the factors was ratio of NaOH to solid (fold, C) > extraction temperature (°C, B) > NaOH concentration (M, A) > extraction time (h, D). The monosaccharide compositions of polysaccharides from *P. odoratum* were rhamnose, mannose, xylose, and arabinose with the molecular ratio of 31.78, 31.89, 11.11, and 1.00, respectively. The reducing power, the 1, 1-diphenyl-2-picryl-hydrazil (DPPH) radical scavenging rate, the hydroxyl radicals scavenging rate, and the inhibition rate to polyunsaturated fatty acid (PUFA) peroxidation of the alkaline soluble polysaccharides from *P. odoratum* at 1 mg/mL were 9.81%, 52.84%, 19.22%, and 19.42% of ascorbic acid at the same concentration, respectively. They also showed antimicrobial activity against pathogenic bacteria *Staphylococcus aureus, Pseudomonas aeruginosa, Bacillus subtilis*, and *Escherichia coli*.

## 1. Introduction

*Polygonatum odoratum *(Mill.) Druce is an edible herb mainly distributed in the Southern area of China and some other south east Asian countries [1], and it is also found to be in Russia and other Europe countries [2, 3]. In particular in Hunan Province of southern China, cultivated* P. odoratum* is customarily called “Xiang Yuzu” by local people, and the dried rhizome is often used as a common Chinese traditional medicinal herb for removing dryness, promoting secretion, and quenching thirst [4]. It is also used as functional foods and traditional Chinese medicines for treatment of diabetes [5]. People are also accustomed to adding the powder made from its dried rhizome to noodles, teas, biscuits, and other health care products or cooking the rhizome with meats and porridges. Generally,* P. odoratum* is now attracting more and more attention for its healthy and medical value.

Many active compounds have been identified in* P. odoratum* such as mucous polysaccharides [6], homoisoflavanones [7], azetidine-2-carboxylic acid [5, 8], saponin [9], and steroidal compounds [10]. Among them, polysaccharides are abundant in* P. odoratum* and considered as one of the important bioactive components with functions of antitumor, antidiabetes, and antioxidation. Extraction of polysaccharides from* P. odoratum* is an important approach to further research or scale application. Tomshich et al. [11] reported extraction and purification of polysaccharides from ten medicinal plants including* P. odoratum*, and several reports were documented on extraction and bioactivities of polysaccharides from* P. odoratum* in recent years [1, 12]. Generally, researches were focused on the study of extraction of neutral soluble polysaccharides from* P. odoratum*, using hot water extraction or hot water extraction assisted by physical technologies such as microwave and ultrasound, to optimize the productivity of polysaccharides. Nevertheless, it should be noted that hot water extraction is often associated with lower leaching efficiency, higher extraction temperature, more time-consuming, and fewer monosaccharide compositions [13]. In our previous experiment, the highest yield of hot water extraction assisted by microwave or ultrasound from* P. odoratum* was only nearly 7%, and three kinds of monosaccharides were detected, suggesting that there were plenty of polysaccharides in extraction residues insoluble in hot water. Kim et al. [14] reported that alkaline extraction polysaccharides from Korean basidiomycetes were composed of four kinds of monosaccharides and displayed antitumor activity. Liu et al. [15] reported that the extraction rate of polysaccharides by mild alkaline hydrolysis from* Ganoderma lucidum *was significantly higher compared with hot water extraction. But there were no reports published about the alkaline extraction of polysaccharides from* P. odoratum* until now.

The objective of the work reported in this paper was to improve polysaccharides yield of alkaline soluble extraction from* P. odoratum* through an orthogonal test, which explored the relationship between four selected variables and determined the optimal variable values to optimize the alkaline extraction conditions, and to further investigate the bioactivity of the alkaline soluble polysaccharides extracts.

## 2. Materials and Methods

### 2.1. Reagents

Ethyl mercaptan, trifluoroacetic acid (TFA), acetic anhydride, pyridine, and 1, 1-diphenyl-2-picryl-hydrazil (DPPH) were from Sigma Co. (St. Louis, USA). Beef extraction peptone medium and LB medium were from Guangrui Biomart Co. (Shanghai, China). All the other chemicals and reagents used were of analytical grade.

### 2.2. Pretreatment of Rhizome of* P. odoratum*

Rhizome of* P. odoratum* was collected in August, 2013, from a natural habitat in Hunan, China, and authenticated by associate Professor Zefa Liu, Loudi Agricultural Institute, Hunan, China. Fresh rhizome was washed, sliced to pieces, and dried at 50°C for 48 h. Then the pieces were ground to powder with a grinding machine. The powder was sieved with a nylon sieve of 0.18 mm diameter grids. 100 g of the sieved powder was mixed with 300 mL petroleum ether and heated at 55°C water bath for 4 h. After filtration, the sediment was dried at 80°C for 12 h, then mixed with 80% ethyl alcohol and heated at a 65°C water bath for 1 h. After filtration again, the sediment was dried at 65°C for 12 h to get powder of* P. odoratum*.

### 2.3. Orthogonal Array Design

According to the previous study of single variable test, four control variables were selected as follows: NaOH concentration (M), extraction temperature (°C), ratio of NaOH to solid (v/w, fold), and extraction time (hours). The variables of extraction optimization design were as shown in Table 1. Rhizome powder (1 g) was used in each test according to a L_9_(3^4^) array. After finishing all the tests, the solution was filtered by vacuum, and 2 M HCl was added to it to adjust pH value to 7.0 and then subjected to 100 mL 95% ethyl alcohol at room temperature for 12 h. After centrifugation at 6,000 ×g for 10 min, the sediment was dissolved in distilled water to determine the yield. The sediment was dried to get the crude polysaccharides and stored at room temperature until use.

### 2.4. Purification of Polysaccharides

0.5 g of crude polysaccharides was dissolved in 5 mL distilled water, added to 5 mL 10% trichloroacetic acid (TCA), and stored overnight at room temperature. After centrifugation at 8,500 ×g for 30 min, the supernatant was treated with 1/5 volume of Sevag reagent [16]. The procedure was repeated at least for 10 times to get the purified polysaccharides. The purity of polysaccharide was determined by using a UVmini-1240 spectrophotometer (Shimadzu Co., Tokyo, Japan). The scan range was 190–350 nm to detect the absorbance of impurities caused by proteins (280 nm) and nucleic acids (260 nm) [17]. The purified polysaccharides were stored at 4°C until use.

### 2.5. Polysaccharide Hydrolysis and Monosaccharide Derivatization

Polysaccharide hydrolysis and derivatization were performed according to the method of Pitthard and Finch [18]. 15 mg of the purified polysaccharides was added to a test tube with 4 mL 3 M TFA and heated at 120°C to hydrolyze for 5 h. After vacuum rotary evaporation, 2 mL ethyl mercaptan and 1 mL TFA were added, stirred for a while, and incubated for 35 min and then evaporated again. The mixture of 2 mL acetic anhydride and 2 mL pyridine was added and heated at 55°C for 4 h. This solution of monosaccharide derivatives was used directly for GC-MS analysis.

### 2.6. GC-MS Analysis

GC-MS detection wascarried out according to Chunsriimyatav et al. [19], using a QP2010 SE gas chromatography-mass spectrometer (Shimadzu Co., Tokyo, Japan) with a HP-5 ms id 30 × 0.32 mm fused-silica column coated with df 0.25 *μ*m film (Agilent Technologies, California, Palo Alto, USA). The split-splitless injector temperature was 260°C. The chromatographic procedures were as follows: initial temperature 80°C for 2 min, up to 210°C at 1°C/min, then up to 280°C at 25°C/min, and kept at 280°C for 6 min. Helium (99.999%) was used as a carrier at 1 mL/min. Electron impact ionisation was 70 eV with 1.5 scans/s at range of* m*/*z 35–600*, and the source temperature was 220°C.

### 2.7. Antioxidant Activity Determination

#### 2.7.1. Reducing Power Assay

The reducing power of the polysaccharides was determined according to the method of Juntachote and Berghofer [20]. 0.5 mL 0.2 M phosphate buffer (pH 6.6) and 1.5 mL 0.3% K_3_Fe(CN)_6_ were added to a test tube and mixed with polysaccharides (1–10 mg/mL). The solution was incubated at 50°C for 20 min, then 1 mL 10% TCA was added, and centrifuged at 3,000 ×g for 10 min. 2 mL of the supernatant was mixed with 0.5 mL 0.3% FeCl_3_ to measure 700 nm absorbance after 10 min.

#### 2.7.2. DPPH Radical Scavenging Rate

Free radical scavenging rate of the polysaccharides was determined according to the method of Chen et al. [21] and Ulbin-Figlewicz et al. [22]. 2 mL of 0.03 mM fresh-prepared DPPH-ethanol solution was mixed with 0.2 mL of the polysaccharides (1–10 mg/mL). The reaction mixture was stirred and immediately incubated at 25°C for 15 min. The reduction of the DPPH free radicals was determined at 517 nm absorbance.

#### 2.7.3. Hydroxyl Radical Scavenging Rate

Hydroxyl radical scavenging rate of the polysaccharides was determined according to Li et al. [23] with minor modification. 0.1 M pH 7.4 phosphate buffer was used as blank. 10 mL reaction mixture contained 5 mL 0.2 M pH 7.4 phosphate buffer, 0.6 mL 5 mM 1, 10-phenanthroline monohydrate, 0.4 mL 7.5 mM FeSO_4_, 0.2 mL 30% H_2_O_2_, 2.8 mL distilled water, and 1.0 mL different concentration of polysaccharides (1–10 mg/mL). The reaction mixture was stirred and immediately incubated at 37°C for 40 min. Absorbance was measured at 510 nm, and the hydroxyl radical scavenging rate was calculated as the following equation:

(1) $$\begin{array}{c} \text{Scavenging}\;\text{rate}\left(\%\right)=\frac{\left[A_{\text{sample}}-A_{\text{damage}}\right]}{\left[A_{\text{nondamage}}-A_{\text{damage}}\right]}, \end{array}$$

where *A*_sample_ was absorbance of mixture containing polysaccharides and H_2_O_2_; *A*_damage_ was absorbance of mixture containing H_2_O_2_; *A*_nondamage_ was absorbance of mixture containing polysaccharides.

#### 2.7.4. Inhibition Rate of Peroxidation of Polyunsaturated Fatty Acid from Lipoprotein

Inhibition rate of polyunsaturated fatty acid (PUFA) peroxidation from lipoprotein was determined according to Zhang et al. [24]. Yolk suspension was prepared with addition of fresh egg yolk to 0.1 M pH 7.4 phosphate buffer (v/v = 1/25). 0.2 mL yolk suspension was mixed with 0.1 mL different concentration of polysaccharides (1–10 mg/mL). 0.2 mL 25 mM FeCl_2_ and 1.5 mL 0.1 M pH 7.4 phosphate buffer were added to the mixture and incubated at 37°C for 15 min with continuous vibration. And then 0.5 mL 20% TCA was added to the mixture and centrifuged at 5,000 ×g for 10 min. 2 mL of the supernatant was added with 1 mL 0.8% thiobarbituric acid, and the solution was incubated at 75°C for 10 min. After cooling to room temperature, absorbance was determined at 532 nm. The inhibition rate was calculated using the following equation:

(2) $$\begin{array}{c} \text{Inhibition}\;\text{rate}\%=\frac{\left[A_{\text{control}}-A_{\text{sample}}\right]}{A_{\text{control}}}, \end{array}$$

where *A*_control_ was absorbance of mixture using the same volume of distilled water instead of polysaccharides; *A*_sample_ was absorbance of mixture containing polysaccharides.

### 2.8. Antimicrobial Activity

The antimicrobial activity was determined following the method of Lipipun et al. [25] with modification. Beef extraction peptone medium and broth medium were prepared and sterilized in an autoclave. Four bacterial strains* Staphylococcus aureus*,* Pseudomonas aeruginosa*,* Bacillus subtilis,* and* Escherichia coli* were individually incubated on LB medium at 37°C for 24 h, and bacterial plaques were selected and suspended to 9 mL sterile saline to prepare bacterial suspension at concentration of 1 × 10^8^ cfu/mL. 0.2 mL bacterial suspension was uniformly coated on beef extraction peptone medium of the petri dishes. Plates of sterile filter paper, 6 mm in diameter each, were soaked in different concentration of polysaccharides (1–10 mg/mL) and dried and then were placed into the petri dishes to incubate at 37°C for 24 h. Result was calculated by measuring the zone of inhibition in millimeters.

### 2.9. Data Analysis

All experiments were repeated at least three times and the results were expressed as mean ± standard error. Statistical analyses were performed using SPSS13.0, and the data were analyzed using one-way ANOVA followed by* LSD* test (*P* < 0.05).

## 3. Results and Discussion 

### 3.1. Alkaline Extraction of Polysaccharides

The factorial design of four factors and three levels was carried out to evaluate the extraction efficiency of polysaccharides. The four factors were NaOH concentration (A), extraction temperature (B), ratio of NaOH to solid (C), and extraction time (D). Therefore, nine experiments of L_9_(3^4^) array were performed, and factors and levels were depicted in Table 1. The results showed that ratio of NaOH to solid (C) was more significant (*R* = 6.35) than the other factors, and the order of the factors was found to be ratio of NaOH to solid (fold, C) > extraction temperature (°C, B) > NaOH concentration (M, A) > extraction time (h, D). The optimal extraction parameters were A1, B3, C1, and D3, namely, NaOH concentration 0.3 M, extraction temperature 80°C, ratio of NaOH to solid 10-fold, and extraction time 4 h, respectively. Under the optimal condition, the extraction was repeated and the actual yield was 17.28% (*n* = 3). Jiang et al. [12] reported that the yield of polysaccharides using hot water extraction (extraction times = 4) was about 14.2%, and Lan et al. [1] reported that its yield using hot water extraction assisted by ultrasound (extraction times = 3) was 15.15%. The yield of alkaline extraction from* P. odoratum* was higher than that of hot water extraction. In addition, this method was much more time-saving because it was needed to be extracted only once.

### 3.2. GC-MS Analysis of Polysaccharides

**Figure 1:**
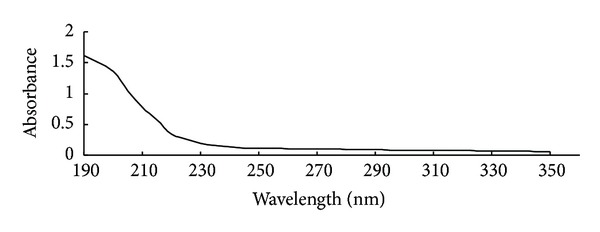
UV spectrophotometry analysis of the purity of alkaline soluble polysaccharides from* P. odoratum.*

TCA and Sevag reagent were employed to get rid of impurities such as proteins and nucleic acids before GC-MS analysis. As shown in Figure 1, there were no absorption peaks at 260 nm and 280 nm, with the absorbance only 0.106 and 0.095, respectively, and an obvious absorbance of polysaccharides at 190 nm was observed 1.621, showing that the polysaccharides were free of impurities such as proteins and nucleic acids and were reliable for further analysis [26].

**Figure 2:**
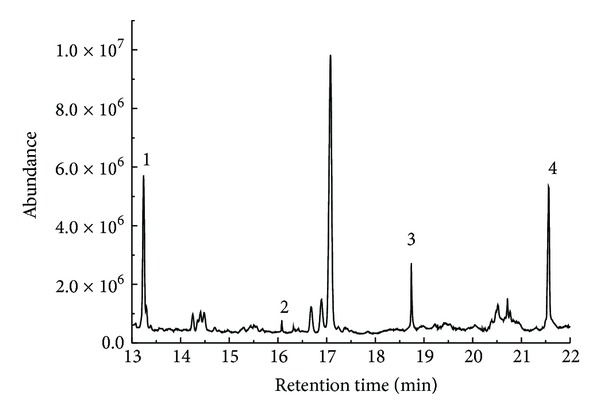
Total ion chromatogram of the peracetylated diethyl dithioacetals of the hydrolysate derivatives of alkaline soluble polysaccharides from* P. odoratum* (1: mannose, 2: arabinose, 3: xylose, and 4: rhamnose).

The polysaccharides were hydrolyzed by TFA and derived by “ethyl mercaptan-acetic anhydride-pyridine” method as mentioned before. The derivatives were used to determine the monosaccharide compositions, because monosaccharide in this condition was converted to volatile substances and easy to be detected by GC-MS [18]. Lan et al. [1] determined three kinds of monosaccharides from neutral soluble polysaccharides in* P. odoratum* by GC, which were fucose, mannose, and galactose with the molecular ratio of 4.72, 3.90, and 1.00, respectively. In our experiment, monosaccharide compositions of alkaline soluble polysaccharides in* P. odoratum* were detected by GC-MS (Figure 2), and the compositions consisted of four kinds of monosaccharide as rhamnose, mannose, xylose, and arabinose with the molecular ratio of nearly 31.78, 31.89, 11.11, and 1.00, respectively (Table 2). The results showed that alkaline soluble polysaccharides differed from neutral soluble polysaccharides in compositions. Tomshich et al. [11] reported that neutral soluble polysaccharides from* P. odoratum* roots with additional ammonium oxalate and NaOH purification steps consisted of arabinose, mannose, and rhamnose with the molecular ratio of 3.38, 3.31, and 1.00, respectively, which were similar to these alkaline soluble polysaccharides in compositions. In addition, Lan et al. [27] reported that two types of dietary* Polygonatum* fibers DFPS and DFDS extracted by NaOH consisted of arabinose, xylose, sorbose, mannose, and galactose with different molecular ratios, which were also similar to the alkaline soluble polysaccharides in compositions. It was indicated that, during the processing method, NaOH might change the chemical structure of polysaccharides.

### 3.3. Antioxidant Activity Analysis

**Figure 3:**
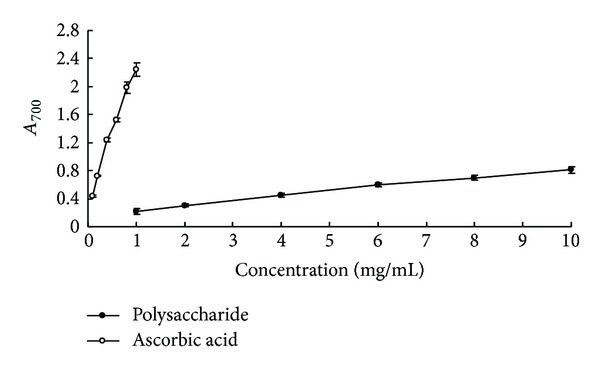
Reducing power of alkaline soluble polysaccharides from* P. odoratum* and ascorbic acid.

The reducing power is frequently used to evaluate the electron donating ability, and there is a close correlation between the antioxidant activity and the reducing power of polysaccharides. Therefore, researchers often use the reducing power as an important standard for evaluating the antioxidant ability [28, 29]. Commonly, the reduction of three-valent iron ion to two-valent iron ion is determined at the absorbance 700 nm, and ascorbic acid is used as control in assay of reduction ability. As shown in Figure 3, the reducing power of alkaline solublepolysaccharides in* P. odoratum* increased as the concentration increased. By comparison, ascorbic acid showed greater reducing power than polysaccharides. The reducing power of 1 mg/mL polysaccharides was 0.22 ± 0.037, about 9.81% of the activity of ascorbic acid at the same concentration, while the reducing power of 1 mg/mL ascorbic acid reached 2.242 ± 0.100. The results indicated that reductone and hydroxide groups of polysaccharides could play a role as electron donors [30]. Nevertheless, the reducing power of polysaccharides was apparently lower than those of ascorbic acid and homoisoflavanones from* P. odoratum* [31], indicating that alkaline soluble polysaccharides from* P. odoratum* were weak electron donors.

**Figure 4:**
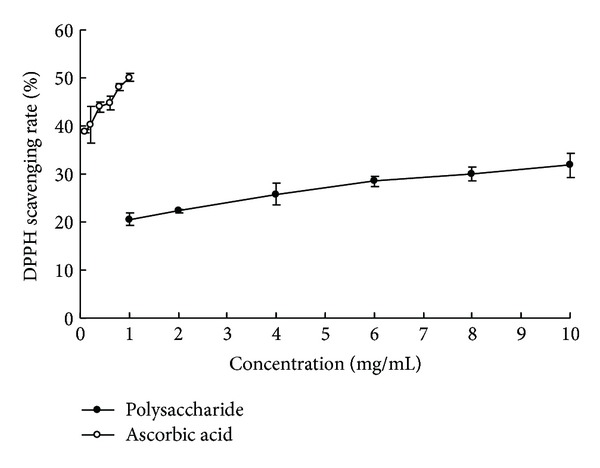
DPPH radical scavenging rate of alkaline soluble polysaccharides from* P. odoratum* and ascorbic acid.

DPPH radical scavenging is a convenient method to rapidly assess the antioxidant ability of biological extracts, because DPPH is a kind of stable nitrogen-centered free radical [32]. It is based on ethanolic DPPH solution from mauve to yellow due to electron donation. Gui and Ryu [28] reported that polysaccharides from plant showed high DPPH scavenging activity. As depicted in Figure 4, a considerable scavenging rate of alkaline soluble polysaccharides in* P. odoratum* was observed. The DPPH scavenging rate of 1 mg/mL polysaccharides was 20.53%, about 40.97% of the activity of ascorbic acid at the same concentration, while the scavenging rate of 1 mg/mL ascorbic acid was 50.11%. The results indicated that alkaline soluble polysaccharides in* P. odoratum *were good DPPH scavengers.

**Figure 5:**
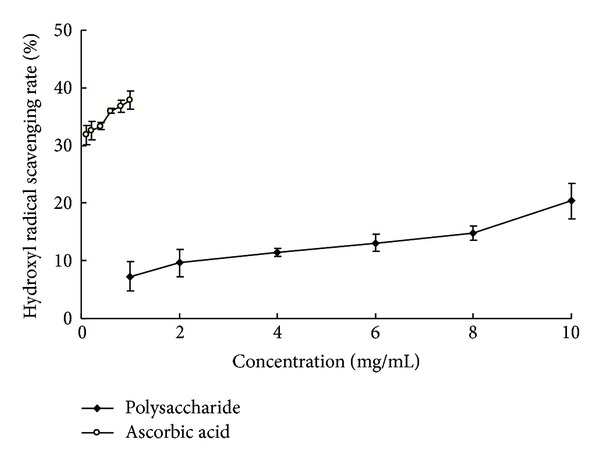
Hydroxyl radical scavenging rate of alkaline soluble polysaccharides from* P. odoratum* and ascorbic acid.

Hydroxyl radicals are the most toxic and active among free radicals. Generally, the scavenging ability of polysaccharides is often evaluated through Fenton reaction [33]. As shown in Figure 5, the hydroxyl radical scavenging rate of alkaline soluble polysaccharides in* P. odoratum* showed an ascending trend as the concentration of polysaccharides increased, but the scavenging rate was only 7.27% at 1 mg/mL. Ascorbic acid is a known strong scavenging agent of hydroxyl radicals, and its scavenging rate at the same concentration was up to 37.82%, while the scavenging rate of 1 mg/mL polysaccharides was only 19.22% of ascorbic acid at the same concentration. The results showed that alkaline soluble polysaccharides in* P. odoratum* were weak hydroxyl radical scavengers.

**Figure 6:**
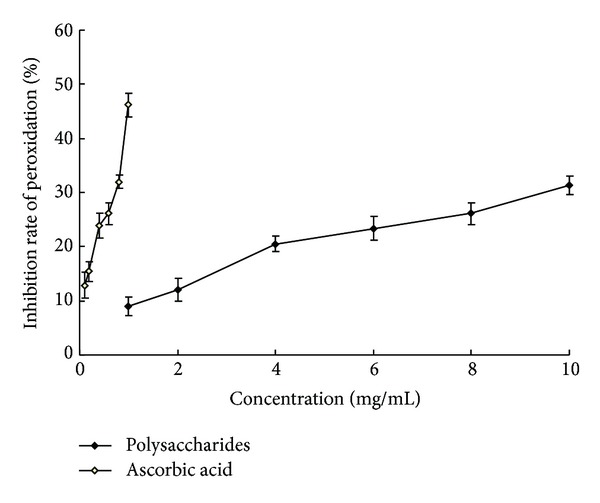
Inhibition rate of PUFA peroxidation of alkaline soluble polysaccharides from* P. odoratum* and ascorbic acid.

PUFA plays an important role in maintaining cell transport system and enzyme activity, and it is easy to be peroxidized [34]. The protective ability of PUFA is used to evaluate the antioxidant activities of polysaccharides, because PUFA from yolk lipoprotein induced by iron is inhibited by polysaccharides. Thus, the antioxidant activity of polysaccharides is correlated with this inhibition rate. The system is applied to determine the activity of effective ingredients of Chinese traditional medicine [24]. As depicted in Figure 6, the inhibition rate of PUFA peroxidation of alkaline soluble polysaccharides in* P. odoratum* was in an ascending trend when the polysaccharides concentration was increased from 1 to 10 mg/mL, but the inhibition rate of polysaccharides was lower compared with ascorbic acid. The inhibition rate of 1 mg/mL polysaccharides was only 19.42% of ascorbic acid at the same concentration.

### 3.4. Antimicrobial Activity

Research on antimicrobial activity of polysaccharides may contribute to valuable new information for future antibiotic development. Many authors have previously investigated the antimicrobial activity of polysaccharides from a wide range of organisms such as seaweeds, mushroom, and durian fruit [25, 35, 36]. In* P. odoratum*, homoisoflavanone, triterpenoids, steroidal saponins, and volatile compounds such as long chain fatty alcohols and phenolic compound were identified and isolated, and these compounds showed high antimicrobial activity against bacteria or fungi [19]. In this study, the highest inhibition zone of 14.93 ± 1.35 mm was observed against* S. aureus*, and the lowest inhibition zone of 10.98 ± 2.15 mm was observed against* B. subtilis *at concentration of 10.0 mg/mL of alkaline soluble polysaccharides. The extracts of alkaline soluble polysaccharides from* P. odoratum* showed antimicrobial activity against all the tested pathogenic bacteria (Table 3). The results enforced the idea that* P. odoratum *is a potential source to be considered for substances in new drug development.

## 4. Conclusion

In the present study, an orthogonal test was carried out to optimize extraction of alkaline soluble polysaccharides from* P. odoratum*. The optimal parameters were NaOH concentration 0.3 M, temperature 80°C, ratio of NaOH to solid 10-fold, and extraction time 4 h, and the actual yield was 17.28%, in which ratio of NaOH to solid (C) was more significant (*R* = 6.35) than the other factors, and the order of the factors was found to be ratio of NaOH to solid (fold, C) > extraction temperature (°C, B) > NaOH concentration (M, A) > extraction time (h, D). The alkaline soluble polysaccharides consisted of four monosaccharide compositions, which were rhamnose, mannose, xylose, and arabinose with the molecular ratio of 31.78, 31.89, 11.11, and 1.00, respectively. They showed antioxidant activity such as DPPH radical scavenging ability, hydroxyl radical scavenging ability, hydroxyl radical scavenging ability, and PUFA protective ability, but the antioxidant activity was lower compared with the same concentration of ascorbic acid. Moreover, the alkaline soluble polysaccharides from* P. odoratum *showed antimicrobial activity against* S. aureus*,* P. aeruginosa*,* B. subtilis,* and* E. coli*.

## Figures and Tables

**Table 1:** Optimization of alkaline extraction parameters of polysaccharides from *P. odoratum*.

Number	NaOH concentration (M)	Extraction temperature (°C)	Ratio of NaOH to solid (v/w)	Extraction time (h)	Extraction yield (%)
1	1 (0.3)	1 (60)	1 (10)	1 (1)	15.11
2	1	2 (70)	2 (15)	2 (2)	14.42
3	1	3 (80)	3 (20)	3 (4)	13.28
4	2 (0.6)	1	2	3	13.16
5	2	2	3	1	8.55
6	2	3	1	2	16.22
7	3 (0.9)	1	3	2	5.65
8	3	2	1	3	15.21
9	3	3	2	1	13.43
*k*_1_	14.27	11.31	15.51	12.36	
*k*_2_	12.64	12.73	13.67	12.10	
*k*_3_	11.43	14.31	9.16	13.96	
*R*	2.84	3.00	6.35	1.86	

**Table 2:** Molecular ratio of monosaccharide mixture from alkaline soluble polysaccharides from *P. odoratum*.

Monosaccharide	Formula	Retention time (min)	Molecular ratio
Rhamnose	C_18_H_30_O_8_S_2_	21.56	31.78
Mannose	C_20_H_32_O_10_S_2_	13.24	31.89
Xylose	C_17_H_28_O_8_S_2_	18.74	11.11
Arabinose	C_17_H_28_O_8_S_2_	16.08	1.00

**Table 3:** Antimicrobial activity of alkaline soluble polysaccharides from *P. odoratum*.

Polysaccharide concentration (mg/mL)	Diameter of inhibition zone			
	*S. aureus *	*P. aeruginosa *	*B. subtilis *	*E. coli *
Blank	—	—	—	—
1.25	7.30 ± 1.18	—	—	—
2.50	8.33 ± 1.53^a^	6.81 ± 1.27^c^	—	7.45 ± 1.32^ab^
5.0	10.38 ± 1.80^a^	7.96 ± 0.79^b^	6.71 ± 0.87^c^	7.73 ± 1.22^b^
7.5	14.32 ± 1.05^a^	9.13 ± 0.83^b^	8.87 ± 1.89^b^	9.38 ± 1.56^b^
10.0	14.93 ± 1.35^a^	11.58 ± 2.21^bc^	10.98 ± 2.15^c^	12.43 ± 1.42^b^

All results were presented as the means of three experiments. Values in the same lines with different letters were significantly different (*P* < 0.05).
